# Notes and News

**Published:** 1904-12-17

**Authors:** 


### Notes and News.

Mrs. Watson Cheyne has arranged a series of dances to be held at the Royal Botanical Gardens in aid of the Removal Fund of King's College Hospital.

We are glad to learn that, after a meeting in the locality on behalf of the North-Eastern Hospital for Children, several handsome donations were promised towards preventing the closing of two of the wards.

The Grand Duchess Vladimir has arranged a concert in St. Petersburg in aid of the Russian wounded. Madame Adelina Patti has gone to St. Petersburg to stay with the Grand Duchess, and will sing at the concert.

At the Conference of the Metropolitan and South-Eastern Poor Law Divisions, held last week, Mrs. Close's proposal to form a scheme to send English children under the Poor Law to Canada was discussed. The general feeling of the meeting was with Mrs. Close, but the Chairman, Mr. T. W. Russell, gave his opinion that the scheme was one that must be left to private endeavour.

The Royal Commission on the Feeble-Minded is now sitting and taking evidence. Miss Dendy, hon. sec. of the Lancashire and Cheshire Society for the Permanent Care of the Feeble Minded, one of the witnesses, on Monday expressed her opinion that there should be statutory prohibition of marriage on the part of the feeble-minded. If carried into effect, this would necessitate the permanent detention of the afflicted persons.

Dr. Doyen, who claims to have discovered the microbe of cancer and a means of combating it, was to make a further communication before the French Academy of Medicine on Thursday. The Committee of the Pasteur Institute and the French College of Surgeons are investigating Dr. Doyen's claim, and Dr. Metchinkoff is himself conducting some of the investigations. It has been stated that favourable opinions have emanated from the institutions referred to, but, as a matter of fact, no opinion of any kind has been yet made public.

Berlin is provided with a new medical journal entitled the *Medicinische Klinik.*

Through the generosity of Sir Donald Currie research work at the Edinburgh University will be greatly assisted. He has presented £25,000 to the University.

The Council of the After-Care Association earnestly appeal for help in money or clothes for poor persons discharged recovered from asylums for the insane, and for donations to carry on and extend the work. Branches of the Association are now being formed in connection with asylums all over the country. Subscriptions or donations thankfully received by the Secretary, H. T. Roxby, Esq., Church House, Dean's Yard, Westminster, S.W.

The representatives of the Dublin medical profession have been received by the Irish Commissioner of National Education, the endeavour of the deputation being to obtain primarily an undertaking that the principles of hygiene should be taught in elementary schools. But Sir Christopher Nixon, one of the deputies, went further than this, and advocated the teaching of hygiene and temperance at the earliest age, with a view to instilling the fact that "cleanliness was next to godliness"; and he advocated an examination in the subject of public health. Dr. Starkie, the Commissioner, in reply, expressed himself in favour of encouraging the dissemination of the knowledge of hygiene, but not in favour of it being treated as a subject for examination.

At an inquest held by Dr. Waldo last Saturday on the body of a Bermondsey labourer who had died of anthrax, contracted from some South African goat-skins which he had handled, Dr. T. M. Legge Home Office Inspector of Factories, called attention to the fact that a serum, which has so far shown good results, is now obtainable for the treatment of anthrax, and Dr. Legge further said that he was prepared to supply any medical man with the serum free of charge. We hope that advantage will be taken of this offer, and that the serum will prove a more certain means of cure than those up to now available. The large number of cases of anthrax which have occurred lately are causing some uneasiness. The majority have come from Bermondsey, and two fatal cases have occurred in Dundee.

The sympathy of the whole nation is with those who are fighting the battle against consumption. The appeal of the Brompton Hospital for Consumption for assistance to continue its good work is not likely to be made in vain. Last year the hospital received within its walls 1,721 in-patients and 12,447 out-patients. The most important adjunct to the work of the hospital is the country home at Chobham Ridges, where the open-air system can be carried on to the best advantage. Consumptives are very costly patients, and generous support from the public is especially needed. The expenditure at Brompton is greater than the income, unfortunately, and Mr W. H. Theobald will receive with gratitude donations and subscriptions which are so much needed, and Christmas gifts too will be very welcome.

